# Indigenous Hepatitis E Virus Genotype 1 Infection, Uruguay

**DOI:** 10.3201/eid2001.131471

**Published:** 2014-01

**Authors:** Santiago Mirazo, Victoria Mainardi, Natalia Ramos, Solange Gerona, Andrea Rocca, Juan Arbiza

**Affiliations:** Facultad de Ciencias, Universidad de la República, Montevideo, Uruguay (S. Mirazo, N. Ramos, J. Arbiza);; Hospital Central de las Fuerzas Armadas, Montevideo, Uruguay (V. Mainardi, S. Gerona, A. Rocca)

**Keywords:** hepatitis E genotype 1, HEV, viruses, indigenous, autochthonous, endemic, nonendemic, hepatitis E virus, Uruguay

**To the Editor:** Hepatitis E virus (HEV) is the only virus in the genus *Hepevirus*, which is the only member of the family *Hepeviridae*. HEV is the causative agent of acute hepatitis E, a moderately severe enteric disease with death rates <4% in the general population and ≈30% in pregnant woman (*1*). The virus is transmitted primarily by the fecal-oral route associated with consumption of contaminated drinking water (*2*). HEV infection is of public health concern because it causes large epidemics and endemic waterborne outbreaks in Asia, Africa, and Latin America (*2*,*3*). During the past decade, an increasing number of locally acquired cases have been reported in industrialized and previously non–HEV-endemic countries, where evidence of zoonotic transmission has been discovered (*4*,*5*).

Human HEV sequences are classified into genotypes 1–4, which are subdivided into subtypes. Genotype 1 was isolated in Asian and African countries and from Cuba and Venezuela in Latin America. Genotype 2 was described in Mexico and Africa. These genotypes have been isolated from human samples and are mostly associated with large epidemics and outbreaks of HEV. Genotype 3 is distributed worldwide, and genotype 4 is found in Asia and central Europe. Genotypes 3 and 4 have been isolated from humans and animal reservoirs, mainly pigs and wild boars, and are commonly found in persons with sporadic acute hepatitis cases (*6*). Thus, zoonotic transmission of HEV also poses a danger to public health

Several countries in South America, including Argentina, Brazil, Bolivia, Venezuela, and Uruguay, have reported the detection and characterization of HEV strains. In these studies, 2 Venezuelan strains were classified within genotype 1; the remaining 70 published strains were indigenous isolates belonging to genotype 3 (*7*). However, little data regarding molecular epidemiology of HEV infection in South America exists. Here, we report the identification and molecular characterization of a genotype 1 strain detected in Uruguay, isolated from a person with locally acquired HEV infection. 

The patient was a male, 26 years of age, and a member of the National Army Force, who had symptoms and paraclinical findings compatible with acute hepatitis: malaise, fever, choluria, jaundice, and elevated levels of bilirubin and liver enzymes. Syphilis, leptospirosis, hepatitis A–C, cytomegalovirus, Epstein-Barr virus, and HIV infection were ruled out by laboratory testing. No risk factor for HEV infection arose from the formal questionnaire, including travel out of Uruguay during the 40 days before the onset of the symptoms. Dietary habits of the patient were also investigated. Shellfish intake and water consumption from potentially contaminated supplies were not reported. 

A blood specimen was tested for IgM and IgG against HEV by using Line immunoassay (recomLine HEV IgG/IgM, Mikrogen Diagnostics, Germany); results were positive. To confirm the presence of HEV, RNA was extracted from 140 μL serum and fecal suspension by using QIAamp Viral RNA Kit (QIAGEN, Hilden, Germany), then retrotranscribed with Superscript II (Life Technologies, Grand island, NY, USA). HEV infection was confirmed by using nested PCR amplification of a conserved sequence within the HEV open reading frame (ORF) 2–3 overlapping region (*8*). For genotyping and phylogenetic analysis, a nested PCR was then performed for the ORF1 RNA dependent RNA polymerase (RdRp) region (*9*). PCR products obtained from serum and fecal samples were cloned into pJET Vector (Thermo Scientific); 3 positive clones for each sample were sequenced. Sequences from 6 clones of both specimens were identical. The isolate, He_Uy 16, was deposited into GenBank under accession no. KF680001.

Phylogenetic reconstruction of the ORF2–3 overlapping region showed that the HEV isolated in Uruguay clustered in genotype 1 with high bootstrap values (Figure, panel A). The same was observed from the phylogenetic reconstruction of the ORF1 RdRp region (Figure, panel B). Furthermore, these analyses revealed a very close relationship of this strain isolated in Uruguay to a strain isolated in India, Yam 67 (99.5% nucleotide identity). Additionally, within the ORF1, the strain shares a high percentage of nucleotide identity (99%–100%) with the genotype 1 strains isolated in Cuba (Cub 11 and 13) and Venezuela (VNZ792), the 2 countries in Latin America in which genotype 1 has been reported to be associated with autochthonous cases. This relationship between these strains from South America and the Yam 67 strain from India warrants further investigation. In Uruguay, we have recently reported the full molecular characterization of a set of HEV strains isolated from patients with 9 sporadic cases in a 2-year period; all strains were genotype 3 (*10*). Here, we describe the detection and phylogenetic analysis of a locally-acquired indigenous case of HEV infection associated with genotype 1 in Uruguay. Although the detection of genotype 1 in 1/10 cases might have occurred by chance, this result supports an endemic circulation of HEV in Uruguay. Data presented here, together with recent advancements in molecular epidemiology of HEV infection in South America, reveal an epidemiologic picture more complex than we initially assumed.

**Figure:**
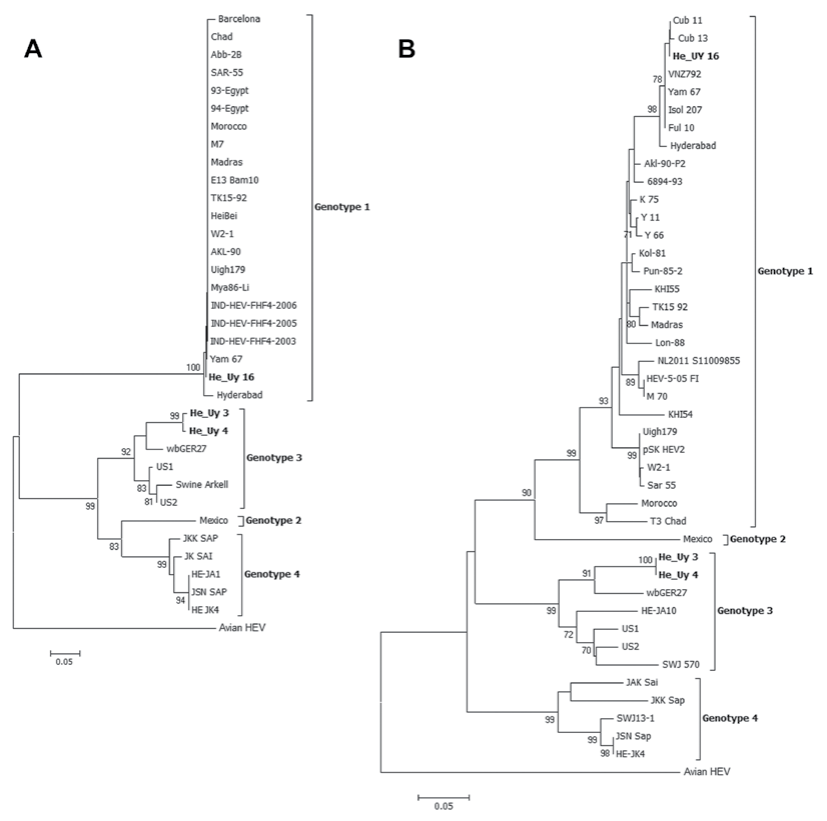
Phylogenetic tree of the Hepatitis E virus constructed on the basis of A) the partial 137-nt open reading frame (ORF) 2–3 overlapping region and B) the 244-nt sequence of the RNA dependent RNA polymerase within ORF1. Trees were generated by using the neighbor-joining method with the Kimura 2-parameter as the substitution model. The robustness of the trees was determined by bootstrap for 1,000 replicates. Values ≥70% are shown. Strains isolated in Uruguay are shown in **boldface**. The isolate of genotype 1 detected in this study (He_Uy 16) clustered (98% bootstrap value) with genotype 1 strains from India and Latin America (Cub 11 and 13 and VNZ792). Scale bars indicate nucleotide substitutions per site.
